# Charitable Appeals and Charities

**Published:** 1889-11-16

**Authors:** 


### The Hospital

A Weekly Institutional Journal of

**Science, Medicine, Nursing, and Philanthropy.**

*For Hospitals, Asylums, Medical Practitioners, Students, Nurses, Families, Parishes, Congregations, and the Charitable Public.*

Vol. VII. No. 164.] [November 16, 1889.

### CHARITABLE APPEALS AND CHARITIES.

Probably not more than one in fifty of the whole population ever gives anything systematically to charities. If we take each family to represent five persons, then not more than one out of every ten householders is a systematic giver, and the rest give nothing at all. Lest this may be considered a hard judgment, we would hasten to point out that in large provincial cities, where we believe many more of the population, in proportion, are regular givers than elsewhere throughout the country, a careful investigation has shown that not one in twenty people ever contributes anything to the support of charities in any form or shape. These facts are accounted for in part by the circumstance that the same people's names are to be found on nearly all subscription lists, whatever may be the object of the appeal, or the nature of the calamity or charity appealed for.

Things being so, what further justification does a writer of an appeal for a worthy object require, for adopting any and every legitimate means in his power, to arouse and fix the attention of some at least of the hundreds of thousands of people, who have never given anything to a charity in their lives? There are, of course, appeals and appeals. Those, on the one hand, which are sound and good in inception and motive, and those which are conceived in a spirit of wantonness or greed and are so hopelessly bad. Mr. Loch, the able secretary of the Charity Organisation Society, has written a long letter to the *Times*, in which he has given some surprising examples of the latter class of appeals. Had he gone a little further, and made it appear that his object was not to discourage sound giving, but to expose shams, we should have been able to welcome his contribution to a difficult question. The facts being as we have stated them, however, we think his letter may do serious injury, not only to the voluntary charities, but to the Charity Organisation Society. It must not be forgotten that, after all, it is the givers who support the Charity Organisation Society, by responding to the appeals which are issued in its behalf from the platform and through the press; and if it were to cease to appeal like other charitable and semi-charitable agencies, it must languish and die for want of money to carry on its work. We have always supported the Charity Organisation Society, though by no means blind to its many failings; but if it is to continue, this society, like its neighbours, whom it is rather fond of calling "suspects," must show a wise discretion in its management and methods of work. It cannot be wise to risk the slaughter of the goose which lays the golden eggs, for the mere sake of a display of knowledge of other people's methods of raising money. We say this because Mr. Loch's letter, however well intended, is likely to cause wide misapprehension, and so to diminish rather than to increase the number of contributors to really-deserving and much-needed charitable institutions and agencies. This would be a real calamity, which we do not believe Mr. Loch intended for a moment, and so we shall hope to see a further and more courageous contribution from his pen, in which he will make his meaning perfectly clear. What we urge him to do is to protect what is good by clearly defining what is bad and unworthy of public support.

Passing on to speak of appeals and appealing, it may be useful to say a word as to the cost of raising funds for charities. It must not be forgotten that the smaller and less known agencies are often at a disadvantage in raising funds, and so it has come to be considered, that such institutions are entitled to spend twice as much on management as their older and bigger rivals. We feel constrained to declare, however, that any charity worthy of the name, ought to be able to raise sufficient income for its purposes by an expenditure of fifteen per cent. and no more of the whole sum raised from voluntary sources each year. In our opinion no committee, whatever be the size of the institution, is justified in paying a single penny more than twenty per cent. of its income from all sources, excluding income from invested property, legacies, and Hospital Sunday and Saturday grants. Twenty per cent. ought to be the maximum, and many charities do not spend more than from seven to ten per cent. upon management, and some even spend much less than this. Hospital secretaries who know their business are specially alive to the importance and justice of our contentions on this point, and deserve public gratitude for the manful way in which they labour to keep down such expenditure at all times. This is abundantly proved by the figures given in *Burdett's Hospital Annual for 1889.* To the man of business as well as to the practical philanthropist this Annual should prove invaluable, because it gives the cost per bed occupied, and the cost of incidental expenses of the principal hospitals in Great Britain and Ireland, so that comparisons are easily made, due regard being had, however, to the considerations urged in the letter-press preceding the tables.

Having said this much about expenditure, we would caution our readers that the very best managed institutions frequently issue the most tasteful and attractive appeals. Such a caution is necessary, because people are apt to think and to say, "What a waste of money it is to spend so "much taste, time, and thought in the preparation and "production of appeal literature. A simple lithographic "circular, costing relatively nothing, would have done "just as well, and would have cost but a fraction of the "sum spent upon this attractive appeal, which is a "veritable work of art." No doubt it frequently is a work of art, and for this very reason it is put aside, carefully examined, and even shown to others by the recipients, instead of being thrown directly into the waste-paper basket. So it happens that one such appeal will produce more money than fifty commoner ones, though they cost twice as much, and the able secretary who puts his brains into his work is worthy of all honour for the care and taste he displays in his appeals.

We hope those who give or who contemplate giving to charities either now, or at Christmas, or at all, will ponder these things. The remedy for the abuses Mr. Loch condemns by implication in his letter to the *Times* is in the hands of the philanthropic public who support any kind of charitable work. It is at least worth while to take a little trouble, to ascertain the merits or otherwise of the particular object which has excited one's interest, before writing a cheque. If found worthy, not only will the pleasure of giving be increased, but, we hope, the amount of the gift also. We have been led to make these observations from the enquiries we receive from charitable people from time to time. Our New Year's Special Number will contain very valuable information about a host of charities, especially about hospitals, and nursing and medical agencies generally. Anyone who will take a little trouble, may, by studying its pages, make a wise selection for their New Year's gifts. The year 1889 has been one of rare prosperity to all classes, and we are confident that this fact must influence for good the volume and character of charitable contributions. There is no greater privilege than that of being able to give to a good object with a free hand. This is especially true, if the donation is made after a conscientious endeavour to ascertain the merits and needs of the institution or person to be benefited. In thanking the noble band of philanthropists who are the mainstay of our voluntary system of charity, we would earnestly invite all non-givers to taste and see how joyful a thing it in reality is, to give liberally of one's abundance, in aid of those, who are, for some inscrutable reason, unable, through no fault of their own, to help or maintain themselves.

